# Health Newscasts for Increasing Influenza Vaccination Coverage: An Inductive Reasoning Game Approach

**DOI:** 10.1371/journal.pone.0028300

**Published:** 2011-12-21

**Authors:** Romulus Breban

**Affiliations:** Unité d'Epidémiologie des Maladies Emergentes, Institut Pasteur, Paris, France; Hungarian Academy of Sciences, Hungary

## Abstract

Both pandemic and seasonal influenza are receiving more attention from mass media than ever before. Topics such as epidemic severity and vaccination are changing the way in which we perceive the utility of disease prevention. Voluntary influenza vaccination has been recently modeled using inductive reasoning games. It has thus been found that severe epidemics may occur because individuals do not vaccinate and, instead, attempt to benefit from the immunity of their peers. Such epidemics could be prevented by voluntary vaccination if incentives were offered. However, a key assumption has been that individuals make vaccination decisions based on whether there was an epidemic each influenza season; no other epidemiological information is available to them. In this work, we relax this assumption and investigate the consequences of making more informed vaccination decisions while no incentives are offered. We obtain three major results. First, individuals will not cooperate enough to constantly prevent influenza epidemics through voluntary vaccination no matter how much they learned about influenza epidemiology. Second, broadcasting epidemiological information richer than whether an epidemic occurred may stabilize the vaccination coverage and suppress severe influenza epidemics. Third, the stable vaccination coverage follows the trend of the perceived benefit of vaccination. However, increasing the amount of epidemiological information released to the public may either increase or decrease the perceived benefit of vaccination. We discuss three scenarios where individuals know, in addition to whether there was an epidemic, (i) the incidence, (ii) the vaccination coverage and (iii) both the incidence and the vaccination coverage, every influenza season. We show that broadcasting both the incidence and the vaccination coverage could yield either better or worse vaccination coverage than broadcasting each piece of information on its own.

## Introduction

The increasing mediatization of medical and epidemiological information determines an increasing role of social behavior for the success of routine vaccination programs. Vaccine promotions, epidemiological newscasts and rumors change the way in which individuals perceive the utility of disease prevention. This phenomenon has been described by empirical studies discussing vaccination against human papillomavirus [1], measles-mumps-rubella [2], [3], poliomyelitis [4] and influenza [5], [6]. Social behavior is particularly important for influenza, which is a seasonal disease and remains a continual epidemic and pandemic threat. In this case, individuals need to make yearly vaccination decisions that are all potentially biased by their perception of costs versus benefits of vaccination.

Mathematical modeling of the potential impact of mass media on the course of epidemics has become a topic of interest only in the past few years [7]–[10]. Several problems have been discussed: sequential disease outbreaks due to the psychological impact of the reported incidence [7], coexistence of multiple endemic states caused by media coverage [8], and the effect of mass media on constant and seasonal vaccination programs [9], [10]. The mathematical techniques utilized so far are compartmental models expressed as systems of ordinary differential equations. However, a technique more adapted to describe decision making and adaptability of individuals in the process of voluntary vaccination is game theory.

Game theory has been successfully applied to modeling the impact of social behavior on vaccination coverage (i.e., the proportion of the population that gets vaccinated). Deductive reasoning games have been used to predict the voluntary vaccination coverage for pathogens that provide permanent immunity [11]–[13]. In the case of pathogens that do not provide permanent immunity (e.g., influenza), several modeling ideas have been studied. An evolutionary game was proposed where an individual copies the vaccination strategy of another with a probability depending on the success of the vaccination strategy [14]. A different approach is that based on inductive reasoning games [15] initially applied to modeling financial markets [16]. In this case, it is conjectured that individuals make repeated vaccination decisions based on their expectations about future epidemics that are, in turn, determined by their collective vaccination coverage. Inductive reasoning games were applied to understanding the dynamics of influenza vaccination coverage assuming both uniform mixing of individuals [17], [18] and mixing through complex contact networks [19].

In this paper, we generalize previous modeling work [17], [18] to study the potential impact of mass media on social behavior and, implicitly, influenza vaccination coverage. So far, inductive reasoning games of influenza vaccination assumed that, at the end of each influenza season, the only epidemiological information available to individuals was whether an epidemic took place. One of the main outcomes of the models with uniform mixing is that influenza epidemics gradually decrease in severity and are occasionally prevented [17]. These models then predict the occurrence of a severe epidemic because individuals expect that their peers will vaccinate and they attempt to free-ride on herd immunity. After the severe epidemic, individuals vaccinate again in increasing numbers, year after year, until an epidemic is prevented and the scenario repeats. Various vaccination incentives were theoretically investigated for their efficiency in preventing severe epidemics [17]. Here we investigate the potential impact of broadcasting various epidemiological information for individuals to evaluate their influenza vaccination decisions at the end of the season. We analyze how epidemiological newscasts may influence the perceived benefit of vaccination, change social behavior, and prevent severe epidemics.

The outline of the paper is as follows. In the next section we introduce our generalized individual-level inductive reasoning game by a set of eight assumptions. It turns out that, in the case of large populations, the mean-field approximation of the game typically provides an adequate description of the coverage dynamics. Hence, a full analysis of the inductive reasoning game is not typically necessary. Then, we develop this approximation in the form of a one-dimensional map where influenza epidemiology and social behavior remain broadly specified by a few unrestrictive axioms. Analysis of this iterated map leads to three key results: (a) individuals will not cooperate enough to consistently prevent influenza epidemics; (b) broadcasting epidemiological information in addition to whether or not an epidemic occurred may stabilize the vaccination coverage and prevent severe epidemics; and (c) the stable vaccination coverage follows the trend of the perceived benefit of vaccination. However, increasing the amount of epidemiological information released to the public may either increase or decrease the perceived benefit of vaccination. To see these results at work, we analyze several model examples. First, we discuss a slight generalization of a previously published model displaying periodically recurring severe epidemics [17], [18] that serves as reference. Then, we discuss two models where individuals know, every influenza season, either (i) the incidence or (ii) the vaccination coverage, in addition to whether there was an epidemic. We show that broadcasting either epidemiological indicator may stabilize the vaccination coverage and prevent severe epidemics. Finally, we discuss two models where individuals know both the incidence and the vaccination coverage every influenza season. One model assumes that individuals are risk-avoiding and use the available information to better protect themselves against infection, while the other assumes that individuals are risk-seeking and use the information to take even greater risk in the attempt to free-ride on herd immunity. We show that broadcasting both the incidence and the vaccination coverage could yield either better or worse vaccination coverage than broadcasting each piece of information on its own. We make concrete assumptions about influenza epidemiology and present numerical results. Finally, we conclude our work.

## Methods

Our model describes a large population of individuals. We account only for the occurrence of epidemics and we do not consider outbreaks since outbreaks become decreasingly important as the population size increases. Influenza transmission models describing large populations [20]–[22] have demonstrated the existence of a *critical coverage level* such that: if the coverage is below the critical level, an epidemic will occur, otherwise the epidemic will be prevented. Our inductive reasoning game includes a simple model of this coverage threshold (see Assumptions q1–2 below), assumes that the vaccine offers complete protection for one year (n.b., increasing the critical vaccination coverage may account for effects of treatment and imperfect vaccines), and proceeds as follows. We consider a large population of individuals acting in their own self-interest. Each individual makes personal decisions as to whether or not get vaccinated against influenza. The collective outcome of these decisions drives influenza epidemiology which, in turn, affects future individual-level decisions. The model proceeds iteratively in two steps per influenza season. The first step is at the beginning of the season when every individual makes their vaccination decision depending on their experience with flu vaccination. An epidemic may occur every influenza season, depending on how the achieved coverage compares with the critical coverage. The second step is at the end of the influenza season when every individual scores their last vaccination decision. We assume that, if they did not get vaccinated, an individual evaluates their decision favorably if they avoided infection (the score has the maximum value of 1) and unfavorably if they got infected (the score has the minimum value of 0). Vaccinated individuals establish the scores of their decisions based on the available epidemiological information. Then, each individual updates their vaccination experience using the score of their last vaccination decision. The whole process repeats in the next influenza season.

### Model definition

For an axiomatic description of the model, we denote the coverage by 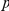, the critical coverage by 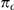, and the probability of getting infected by 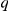. We now present the assumptions that define our generalized inductive reasoning game in mathematical form.

#### Assumption 1

We consider a number of 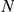 individuals that make yearly vaccination decisions. The interest of the individuals is to avoid getting infected, preferably without having to vaccinate. They act in their own interest and do not communicate their vaccination decisions to each other.

#### Assumption 2

To make their vaccination decision, each individual uses their past experience of vaccination outcomes. Thus, individuals independently decide whether or not to vaccinate using inductive reasoning.

#### Assumption 3

An individual weights their previous vaccination outcomes with respect to their most recent vaccination outcome. A parameter 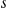 discounts the previous year's vaccination outcome with respect to the outcome of the present year (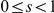). For 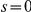, individuals completely ignore the outcome of previous seasons and, as a consequence, do not use inductive reasoning. If 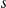 were equal to 1, individuals would not discount the previous vaccination seasons; therefore, the vaccination outcome of the present season (i.e., season 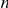) would be as important as any of the previous seasons.

#### Assumption 4

We define a vaccination decision as a realization 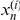 of a Bernoulli variable with parameter 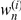 that further depends on a variable 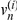. 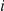 and 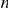 are positive integers; 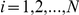 labels the individual and 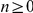 labels the season. If individual 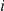 decides to get vaccinated in season 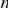 then 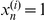, otherwise 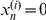. 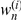 is the probability that individual 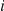 vaccinates in season 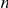. The variable 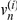 characterizes the *pro-vaccination experience* of the 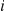
th individual (see details in Assumption 7) and determines 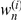. The domains of the variables are as follows: 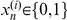, 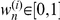, and 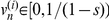.

#### Assumption 5

In year 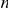, a set of 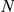 vaccination decisions is made 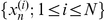 that, together with the pro-vaccination experiences in year 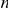, determine the pro-vaccination experiences of all individuals in year 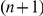, 
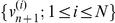
, which further determine 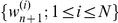, the parameters of the Bernoulli variables in year 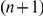. Then, the set of vaccination decisions in year 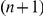 is obtained 
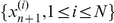
. Our inductive reasoning game is an array of sets of vaccination decisions.

#### Assumption 6

The infection event of individual 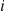 in year 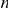 is described by a variable 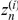. (If individual 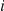 got infected in season 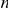 then 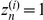, otherwise 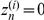.) The infection process is as follows. If 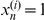 then 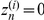. If 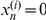, then 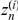 is a realization of a Bernoulli variable with parameter 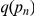, where 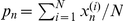 is the coverage achieved that year. That is, if individuals vaccinate, they are fully protected, otherwise they risk infection with probability 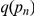.

#### Assumption 7

At the end of the influenza season of year 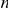, each individual provides a score between 0 and 1 for their vaccination decision 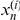 based on their infection status 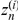 and broadly available epidemiological information. We have three cases: (a1) if 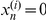 and 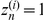 then the score is 1 and 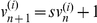; that is, if individual 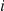 did not get vaccinated and got infected, then the individual considers that the vaccination was necessary; (a2) if 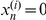 and 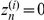 then the score is 0 and 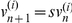; which means that if individual 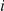 did not get vaccinated and did not get infected, then they consider that the vaccination was unnecessary and (b) if 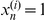, then the score of the vaccination decision is 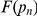 and 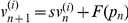. That is, if individual 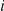 got vaccinated then they did not get infected and use the broadcast epidemiological information to evaluate their vaccination decision (see discussion below);

#### Assumption 8

The probability that an individual chooses to get vaccinated is updated as follows
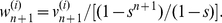
(1)That is, an individual's probability to get vaccinated in the next season is given by the updated cumulative vaccination experience. We have normalized 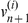 by 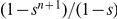 because this factor is the maximum possible value for 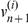 if individual 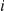 would have fully benefited from vaccination in all of the 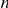 influenza seasons.

We further introduce general assumptions for the functions 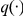 and 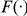 that result from their biological and sociological meaning. The function 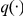 represents the probability of getting infected versus coverage and must illustrate both a critical coverage and a herd immunity effect (i.e., higher overall coverage must offer higher overall protection). We express this mathematically by the following two assumptions.

#### Assumption q1


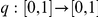
 is continuous. We also require that 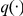 is differentiable everywhere in the domain except at 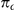.

#### Assumption q2


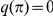
 for 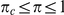 and 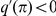 for 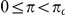, where the prime denotes the derivative with respect to the argument.

We note that the 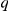-functions obtained from analysis of the Susceptible-Infected-Recoved and Susceptible-Exposed-Infected-Recoved models [17] are compatible with the above assumptions.

A vaccinated individual evaluates their vaccination decision depending on the epidemiological outcome of the influenza season which we express in terms of the vaccination coverage. We make assumptions on the analytic form of 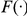 to reflect the fact that the individual tries to benefit from herd immunity and that they are not satisfied to have had vaccinated when epidemics were prevented.

#### Assumption F1


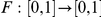
 is continuous and differentiable everywhere in the domain except at 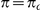.

#### Assumption F2


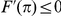
 wherever 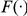 is differentiable. That is, individuals would try to benefit from herd immunity; as coverage increases, the pro-vaccination experience gained by individuals who got vaccinated decreases.

#### Assumption F3


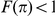
 for 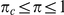. That is, individuals are not fully satisfied to have had vaccinated when epidemics were prevented.

The score function 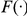 may be interpreted as the *perceived benefit of vaccination*, normalized between 0 and 1. It depends both on the epidemiological information available to vaccinated individuals and how they react to this information. Such a function could be grounded in terms of how individuals seek to maximize their utility, given their estimates of infection risk. We take the function as a given, and note that Assumptions F1–3 are plausible for any underlying model of self-interested behavior. To address risk-avoiding versus risk-seeking vaccination strategies, we discuss score functions in Sec. that combine distinct pieces of epidemiological information (infection incidence and vaccination coverage) in two different ways.

### Mean-field approximation

We now derive a deterministic approximation for the vaccination coverage dynamics in the limit of a large population (i.e., 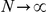). We denote by 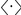 the average over the realizations of the game and introduce the variable 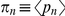 for the average coverage over the realizations of the game in the limit of large 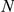. By the Central Limit Theorem (using the Lyapunov condition), we have that 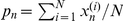 is normally distributed with average 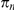 and standard deviation 
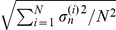
, where 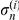 is the standard deviation of the distribution of 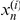 (i.e., 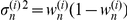). Since 
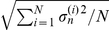
 is bounded from above, the dynamics of 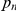 at large finite 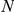 can be approximated by adding Gaussian noise with amplitude 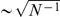 to the dynamics of 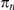. However, in most of the phase space of 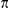, the noise will not change the qualitative dynamics of the orbit and mean-field will be a suitable approximation. Furthermore, since the noise amplitude is small and the functions 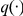 and 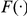 are continuous, we have 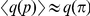 and 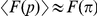.

From the definition of 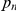, we immediately obtain 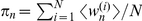. Now, following Assumption 7, it is straightforward to arrive at the equations listed below according to the scoring tree for vaccination decisions
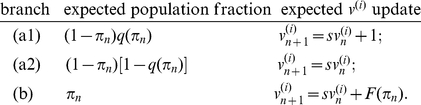
(2)The weighted average of Eqs. (2) yields
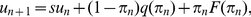
(3)where 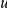 denotes the average of 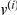 over the entire population. Taking the population average of Eq. (1), we obtain 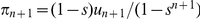, which in the limit of 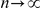 yields simply
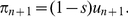
(4)Combining Eqs. (3) and (4), we obtain a mean-field autonomous approximation of the coverage dynamics of the inductive reasoning game without regard to the individual-level processes
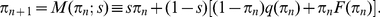
(5)Our dynamical system, 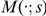, is defined on the unit interval.

### Epidemiological indicators

It is straightforward to relate epidemiological variables to the framework of the mean-field model. We introduce 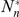 for the number of susceptible individuals in season 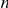 and 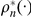 for its distribution over realizations of the game. The incidence in one influenza season is defined as the number of new cases per susceptible individual. Since we model the number of cases among the susceptible individuals by a binomial distribution with parameter 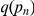 (Assumption 6) and 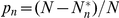, the expected incidence in season 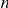, denoted by 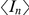, is given by
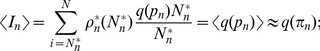
(6)i.e., the probability that an unvaccinated individual becomes infected. Prevalence is defined as the fraction of cases in the general population. The expected prevalence in season 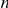, denoted by 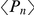, can be written as
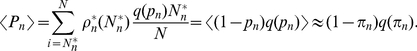
(7)Other epidemiological indicators may be derived in a similar fashion.

## Results

### General results

We first derive three results that apply to all models satisfying Assumptions 1–8, q1–2 and F1–3.

#### Proposition 1

The mean-field model has no attractor included in the 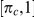 interval.

#### Proof

In the 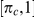 interval, the mean-field model is given by
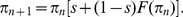
(8)Since 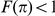 for 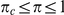 (Assumption F3) we obtain that 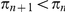. Thus, the 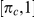 interval is repelling. An orbit starting in the 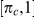 interval decreases monotonically until an iterate belongs to the 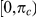 interval.



#### Remark 1-1

The 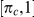 interval may contain points that belong to an attractor of the mean-field model.

These results have important consequences for public health. They demonstrate that, under very general assumptions, a group of self-interested individuals will not cooperate enough to *consistently* prevent influenza epidemics through voluntary recurrent vaccinations. Furthermore, if the score function fulfills assumptions F1–3, even a public health program that manages to increase the perceived benefits of vaccination would not eliminate influenza epidemics. However, a public health program may successfully control the influenza coverage dynamics to achieve more modest goals such as maintaining a high time-average of the coverage and/or a stable coverage. In this study we address the goal of stabilizing the vaccination coverage close to, yet below the critical level. We thus proceed with the fixed-point analysis of the mean-field model given by Eq. (5) in the range where 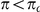 and epidemics are not prevented.

#### Proposition 2

The fixed point of the mean-field model, denoted by 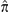, has the following properties:

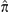
 is the unique solution in the 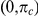 interval of the equation
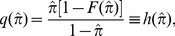
(9)and does not depend on the memory parameter 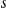.
2. The stability of 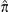 is given by the condition
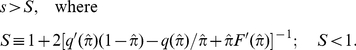
(10)Furthermore, assuming that the functions 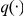 and 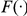 are three times differentiable in the 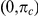 interval, 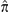 typically loses stability through a period-doubling bifurcation.

#### Proof

1. The equation for 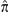 is immediately obtained by setting the fixed-point condition for Eq. (5); i.e., 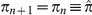. Using Assumptions F1–3 we immediately have that 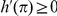 in the 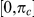 interval, 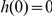 and 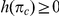. Using also Assumptions q1–2, Eq. (9) has a unique solution in the 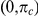 interval. Given that Eq. (9) is independent of 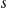, 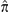 is independent of 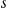, as well.
2. Let us denote by 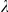 the derivative of the mean-field map at the fixed point 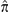; 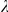 is given by
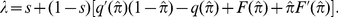
(11)The fixed point 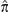 is linearly stable if and only if 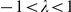 (Ref. [23], Chapter 4). Using Eq. (9) to substitute 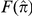 and the Assumptions q1–2 and F1–3, we obtain straightforwardly that 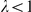 is always satisfied and the condition 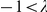 can be written in the form of the Eq. (10). Note that 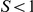 since the square bracket in the definition of 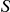 is strictly negative. Thus, the fixed point 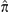 always has a nonempty domain of stability in the parameter space, 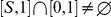. The fixed point 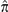 may only lose stability as 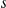 decreases below 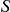; i.e., 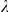 decreases below minus one. With the additional requirements
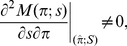
(12)

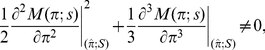
(13)the dynamical system 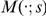 satisfies the conditions for having a generic period-doubling bifurcation at 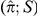 – Ref. [23], Chapter 4, Theorem 4.3 and Ref. [24], Chapter 2.3. Other bifurcations are possible, depending on the particular choices for the functions 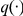 and 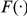. However, they are not generic. In other words, a smooth arbitrary small perturbation of these functions will break the bifurcation into generic ones that survive such perturbations. Equation (12) can be rewritten as
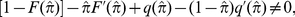
(14)and is always satisfied virtue of Assumptions F1–3 and the fact that 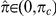. Equation (13) requires that the functions 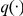 and 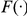 are three times differentiable in the 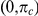 interval and is typically satisfied; i.e., if violated, a smooth arbitrary small perturbation of these functions will have Eq. (13) satisfied.

We note that the period doubling bifurcation can be either *direct* or *inverse* (Ref. [24], Chapter 2.3). In the case of a direct bifurcation, the fixed point 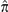 loses stability at 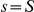 and, simultaneously, a period-two attractor is created. In the vicinity of the bifurcation, the period-two orbit is symmetric about 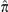, having one iterate above and one below the fixed point. Hence, mild and severe influenza epidemics alternate. We consider this scenario to be an inferior outcome since the health care system has to manage more severe epidemics while the average coverage remains the same. In the case of an inverse bifurcation, the stable fixed point 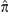 merges with an unstable period-two orbit and loses stability at 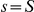. Past bifurcation, the local dynamical structure consists of the unstable fixed point 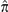 without any attractor in the neighborhood. Hence, an orbit in this region will evolve to some distant attractor. A class of such attractors is created through codimension-one border-collision bifurcations. Due to the discontinuity of the model derivative at 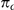, critical periodic orbits with points in the 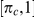 interval are created at particular values of 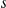. Denoting the 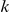
th iterate of the model map by 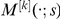, the equation of a period-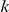 critical orbit is 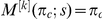. The critical periodic orbit may turn into a noise-robust attractor having at least one point in the 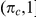 interval; see also discussion in Ref. [18]. If the fixed point 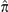 loses stability through an inverse period doubling bifurcation and is captured by such an attractor, then complex coverage dynamics ensues where influenza epidemics are occasionally prevented.

Finally, we present a variational result that links changes in the score function to changes in the stationary coverage.

#### Proposition 3

Let 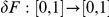 be an infinitesimal deformation of 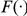 such that the function 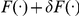 satisfies Assumptions F1–3. If the function 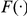 is perturbed by the amount 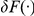, then the fixed point 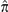 is perturbed by an amount 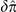 having the same sign as 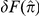.

#### Proof

Applying Eq. (9) for the functions 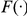 and 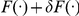, and subtracting, we obtain in the first infinitesimal order
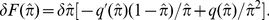
(15)According to Assumptions q1–2, the factor in the square bracket of Eq. (15) is positive.

#### Remark 3-1

The above result can be immediately generalized for finite deformations of 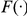 that are performed through sequential infinitesimal steps.

#### Remark 3-2

Stability of the fixed point 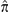 may be lost due to deformations because the stability condition (10) depends on 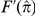.

The above results show that the stable vaccination coverage follows the trend of the perceived benefit of vaccination (i.e., score function 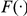). Providing more epidemiological information to the public may cause either an increase or a decrease in the perceived benefit of vaccination, as we show by analyzing two different strategies. Risk-avoiding individuals may use the additional information to avoid infection risk and increase the value of their score function. In contrast, risk-seeking individuals may perceive a lower benefit to vaccination and attempt even more to free-ride on herd immunity. Hence, broadcasting additional epidemiological information may result in either increasing or decreasing the stable vaccination coverage.

### Model examples

To illustrate the results expressed by Propositions 1–3, we discuss and compare five models. We use subscripts 1–3, 4a, 4b to refer to the dynamical elements corresponding to the models 1–3, 4a, 4b, respectively. In each case, we assume that individuals know, at the end of every influenza season, whether an epidemic took place. In particular, for model 1, we assume that this is the only information available, determining a certain form for the function 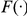. In fact, model 1 is only a slight generalization of the *basic* model previously studied [17], [18] and serves as reference. For the models 2, 3, 4a and 4b we assume that individuals are given additional epidemiological information that determine other functional forms for 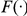, in each case. For the time being, we leave the function 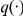 unspecified. Later we discuss a choice of function for 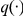 and present numerical results.

### Model 1

In this model, at the end of each influenza season, individuals know only whether there was an epidemic and vaccinated individuals consider vaccination to have been worthwhile only if there was an epidemic. The 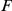-function is given by [18]

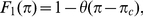
(16)where 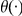 is the unit step function defined as
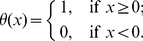
(17)In Ref. [18], 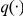 is a piecewise linear function. However, to recover the qualitative features of the dynamics, it is sufficient that 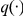 satisfies Assumptions q1–2. Performing a similar analysis, we immediately obtain that there are no fixed points and 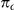 may be attracting only from the left. The coverage dynamics around 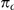 proceeds in a cyclic fashion. When the coverage is less than 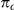, individuals become infected and they increase their probability of getting vaccinated in the next influenza season. The vaccination coverage gradually approaches 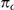, until it eventually slightly exceeds the critical level due to the stochastic nature of the individual-level adaptive decision-making process. At this point, an influenza epidemic does not occur. However, in the following season, many individuals decide not to get vaccinated, as an epidemic did not occur in the previous season; thus vaccination coverage abruptly decreases in the vicinity of 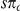 and a severe epidemic ensues. Then, the vaccination coverage repeats in a similar cyclic dynamic. In technical terms, the mean-field dynamics approaching 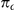 is not robust to noise. Once the coverage slightly exceeds 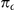 due to noise, the next iterate of the coverage drops in the vicinity of 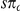 and a severe epidemic occurs [18]. This model is not generic since an infinitesimal perturbation of 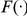 may shift the attractor away from the boundary, destroying the sensitivity to noise. However, the biological and sociological considerations leading to the particular form of the function 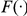 are natural. Consequently, the model may be considered representative for this application. A similar situation occurs for models of disease transmission based on ordinary differential equations. Although the transcritical bifurcation that models the epidemic threshold is not generic, it is considered representative assuming no migration of infected individuals.

Stable, noise-robust, fixed-point dynamics prevent the severe epidemics found in model 1, if and only if 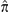, the fixed point of the model under consideration, satisfies the condition
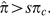
(18)Combining this relation with the stability condition (10) we obtain that the fixed-point dynamics of the vaccination coverage prevent the severe epidemics in model 1 if and only if 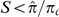. Furthermore, the memory parameter 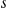 must belong to the following interval
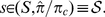
(19)


### Model 2

**Figure 1:**
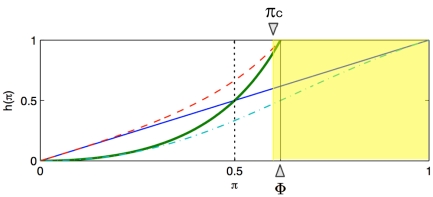
Representation of the possible fixed point equations for the example models. For illustration, we consider 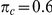 [17], [18]. We assume that 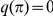 in the shaded region (i.e., where 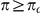) and that 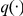 is larger than zero and strictly decreasing in the complementary region. Model 1 has an attractor from the left at 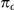. For models 2, 3, 4a and 4b, the functions 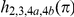 are represented with thin violet continuous, thick green continuous, cyan dot-dashed and red dashed lines, respectively. Note that 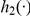 and 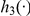 intersect at 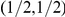, 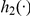 and 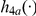 intersect at 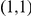, and 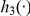 and 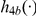 intersect at 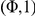, where 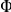 is the golden ratio conjugate. The function 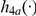 is smaller than 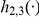 in the unit interval; thus, 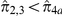. When restricted to the unit square, 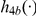 is larger than 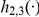; thus, 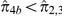.

We consider that, at the end of every influenza season, the individuals know the incidence of infection that was realized in that season.This implies that they also know whether there was an epidemic: if the incidence was larger than zero then there was an epidemic; otherwise there was not. If they got vaccinated, then individuals use the epidemiological information to evaluate their vaccination decisions. In particular, we assume that the score of their last vaccination decision equals the seasonal incidence which also represents the individual-level risk of becoming infected if unvaccinated; see Eq. (6)
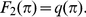
(20)We note that other models are possible. For example, *more concerned* individuals may use a score function that increases faster with the incidence; e.g., 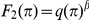, where 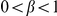. Here we choose 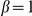 for analytical tractability. Hence, the fixed point equation (9) becomes
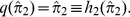
(21)In this case, 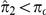 (see Fig. 1) and noise-robust equilibrium dynamics of the coverage is possible. The stability bound for 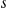 is
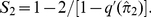
(22)


### Model 3

In this case we assume that, at the end of each influenza season, the vaccination coverage is disclosed to the public. Additionally, we assume that individuals know whether there was an epidemic. An 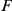-function that summarizes this facts is
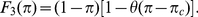
(23)If the epidemic was prevented, then the individuals that got vaccinated evaluate their last vaccination as unnecessary; i.e., 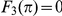, if 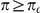. Otherwise, they evaluate their last vaccination as necessary to the degree that their peers did not get vaccinated; 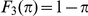, if 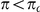. These simple assumptions yield in fact a score function 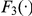 that is discontinuous at 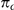 and violates Assumption F1; see Eq. (23). Hence, the mean-field approximation of the inductive reasoning game fails for coverage dynamics in the vicinity of 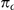. However, the fixed point equation (9) is
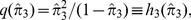
(24)and 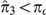 (see Fig. 1). Therefore, the fixed point dynamics of the one-dimensional map does approximate that of the inductive reasoning game. Noise-robust equilibrium dynamics of the coverage is possible. The stability bound for 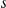 is
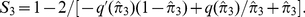
(25)


### Model 4a

Finally, we discuss the case where both incidence and coverage are disclosed to the public. First, we assume that the individuals are avoiding risk of infection by increasing their vaccination scores. In particular, we assume that vaccinated individuals acknowledge a posteriori the benefit of their last vaccination not only for eliminating their personal risk of infection 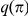 but also for contributing to the proportion of successful free-riders 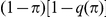. We consider the following scoring function 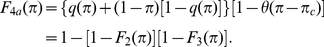
(26)Note that 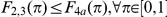. The function 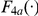 is discontinuous at 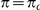; model 4a is subject to the same caveat as model 3 regarding the dynamics in the vicinity of 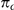. The fixed point equation (9) becomes
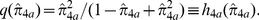
(27)Noise-robust equilibrium dynamics of the coverage is possible since 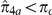 (see Fig. 1). The stability bound for 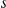 is
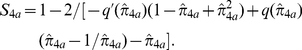
(28)


### Model 4b

In this model, as well, incidence and coverage are disclosed to the public. We assume that the individuals are risk-seeking in their attempt to ride on herd immunity. If they got vaccinated, they evaluate their last vaccination necessary only to the degree that their peers got infected, using a scoring function that equals the prevalence
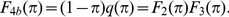
(29)

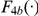
 may also be considered reasonable for the case where just the prevalence of infection is broadcast. Note that 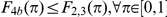. The fixed point equation (9) becomes
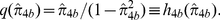
(30)Once again, noise-robust equilibrium dynamics of the coverage is possible since 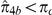 (see Fig. 1). The stability bound for 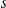 is
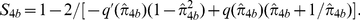
(31)


In Fig. 1, we illustrate the functions 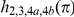 and compare all the models presented above. The relevant domain is the unit square; i.e., 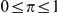 and 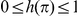. It is straightforward to show that

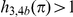
, 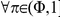, where 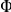 is the golden ratio conjugate;

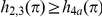
, 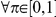, thus 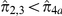;

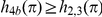
, 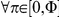, thus 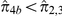.

Thus, among all the models we considered, the ones where individuals know most about influenza epidemiology (i.e., models 4a and 4b) yield the highest and the lowest value for the fixed point of the voluntary vaccination coverage. A comparison between 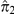 and 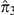 awaits a particular choice for the function 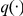.

### Numerics

**Figure 2:**
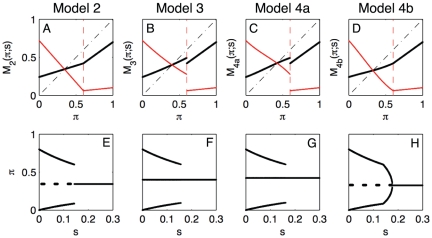
Maps and bifurcation diagrams for the example models 2, 3 and 4a and 4b with the 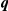-function given by Eq. (32) having 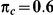 and 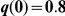 [17], [18]. Panels A and E refer to model 2; panels B and F refer to model 3; panels C and G refer to model 4a; and panels D and H refer to model 4b. Panels A–D represent the model maps 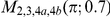 (thick black line) and 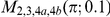 (thick red line) versus 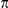, respectively; the thin dot-dashed black line bisects the first quadrant and dashed red vertical line marks 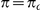. Panels E–H represent bifurcation diagrams of the maps 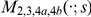 versus 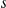, respectively. Continuous lines show how attractors change versus 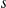 while dashed lines show how unstable fixed points change versus 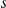.

For a numerical comparison, we have chosen the 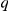-function below [17], [18]

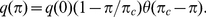
(32)We do not present numerics for model 1 since they have been extensively studied [17], [18]. Figure 2 presents maps and bifurcation diagrams for the other four models where we have chosen 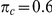 and 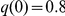, as in Refs. [17], [18]. Each column of panels corresponds to one model: the first column (i.e., panels A and E) corresponds to model 2, the second column (i.e., panels B and F) corresponds to model 3, the third column (i.e., panels C and G) corresponds to model 4a, and the fourth column (i.e., panels D and H) corresponds to model 4b. Panels A–D represent the model maps 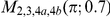 (thick black line) and 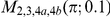 (thick red line) versus 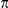, respectively. Panels D–H represent bifurcation diagrams of the maps 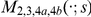 versus 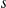, respectively. Several comments are in order.

1. The fixed points 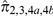 are less than 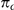 (Fig. 2A–D) and remain constant for changing values of 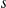 (Fig. 2E–H).
2. The bifurcation structure of 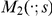 is not generic because 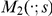 is not generic. In fact, since 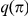 is piecewise linear, 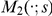 is piecewise linear, as well (Fig. 2A). The fixed point 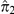 does not undergo a generic period doubling bifurcation (Proposition 2) because a period two critical orbit becomes an attractor at the same parameter value where 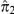 loses stability, 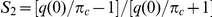 (Fig. 2E). This bifurcation structure does not survive smooth arbitrary small perturbations (Proposition 2).
3. The fixed points 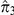 and 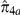 do not lose stability for 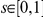 because 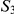 and 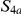 are negative.
4. The fixed point 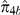 loses stability through a direct period doubling bifurcation.
5. The relations 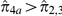 and 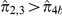 hold, as expected from the general theory.
6. Although 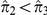, this is not generally true; it is due to the fact that 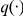 is piecewise linear; see Fig. 1.

**Figure 3:**
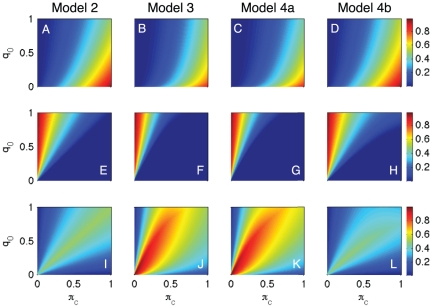
Numerical comparison between the fixed point structure of the example models 2, 3, 4a and 4b with the choice of 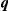-function given by Eq. (32). We explored the parameter space 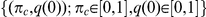. Panels A, E and I refer to model 2; panels B, F and J refer to model 3; panels C, G and K refer to model 4a; and panels D, H and L refer to model 4b. Panels A–D represent how far is the fixed point of each model from the critical vaccination coverage; i.e., 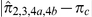, respectively. Panels E–H represent the lower bound of the memory parameter 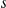 such that the fixed point of the corresponding model is stable; i.e., 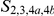, respectively. Panels I–L represent the width of the 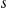-interval where the fixed point dynamics has coverage above 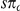; i.e., 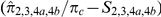, respectively.

Figure 3 presents numerical results for the parameter space 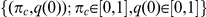. Many features in Fig. 3 can be addressed analytically; we leave the details to the reader. As in Fig. 2, each column of panels corresponds to one model: the first column (i.e., panels A, E, and I) corresponds to model 2, the second column (i.e., panels B, F, J) corresponds to model 3, the third column (i.e., panels C, G and K) corresponds to model 4a, and the fourth column (i.e., panels D, H and L) corresponds to model 4b. Panels A–D show how far the fixed point of each model (i.e., 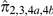) is from the critical vaccination coverage 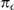. We note that 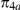 is closest to 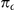, 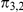 come next (in order) and 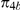 last. Panels E–H plot colormaps for the lower bounds of the intervals of 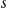 such that 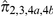 are stable (denoted by 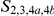), respectively. We remark that 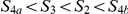; therefore the stability interval is largest for model 4a, then model 3, model 2 and last, model 4b. Finally, panels I–L represent how the sizes of the intervals 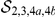 vary with 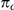 and 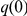, respectively. We notice that 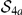 is the largest across all of the domain, followed (in order) by 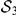, 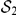 and 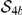.

A model describing a convenient scenario would have the fixed point coverage close to the critical level, a low value of 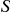 so fixed point stability is achieved even though individuals do not remember many past vaccination outcomes, and a broad 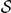-interval for model robustness. Among the models presented here, model 4a performs best, then model 3, model 2 and last model 4b.

## Discussion

Field studies provide evidence of free-riding on influenza herd immunity. For example, *“self-other” optimistic bias* about influenza risk was demonstrated in a sample of New Jersey adults where three out of four individuals estimated their risk of infection below average [27]. Using game theory, it was previously shown that, in the case where individuals know only whether an epidemic takes place every influenza season, free-riding on herd immunity may cause severe drops in the vaccination coverage, leading to major epidemics [17], [18]. Furthermore, these major epidemics could be suppressed by a public health program offering free vaccination for a number of years to every individual, every time the individual decided to get vaccinated [17]. However, other means of epidemic control may be investigated. In a society where vaccines are rarely in short supply, the success of voluntary vaccination programs depends very much on the perceived benefit of vaccination. Mass media is an important factor in shaping social trends and its reach has never been broader. In this work we investigated the potential impact of epidemiological newscasts on the dynamics of voluntary vaccination coverage against seasonal influenza infections.

Our first result addresses the possibility of eliminating influenza epidemics by increasing the vaccination coverage. Recently, the seasonal influenza vaccination coverage has steadily increased in the developed world. The average in five European countries reached 25.9% in 2007–2008 [25], while in the US the coverage reached 41.1% in 2009–2010 [26]. Still, these figures fall short of the critical vaccination coverage, estimated to be in the range of 50–70%; see [17] and references therein. Will the vaccination trend continue such that the coverage exceeds the critical level year after year, eliminating influenza epidemics? The answer is most likely no. If the coverage approached the critical value, epidemic severity would decline and individuals would no longer perceive the same benefit in vaccination, instead attempting to free-ride on herd immunity. Hence, epidemics would persist. Furthermore, our study suggests that mass media would not be able to change this course of events, no matter what epidemiological information were released to the public to change its perceived benefit of vaccination (Proposition 1).

Our second result shows that newscasts providing the individuals with more epidemiological information (e.g., incidence, coverage, or both) may lead to a stable coverage dynamics, suppressing severe epidemics caused by free-riding of herd immunity (Proposition 2). Hence, mass media could effectively be used for public health just as incentives providing free vaccination [17]. The practical implications of this result may be profound as newscasts require no compliance from participants and less logistics than implementation of vaccination incentives.

Our third and last result states that the stabilized level of the vaccination coverage follows the trend of the perceived vaccination benefits (Proposition 3). Still, as they learn more about seasonal influenza epidemiology, individuals have two major choices in changing their vaccination strategies. First, they may assign more utility to vaccination, increasing the overall vaccination coverage. Second, they may take even more risk to free-ride on herd immunity, causing a decrease in the vaccination coverage. Which of these two choices would likely be realized is a difficult question. Using reasonable assumptions, our example models and numerics show that the vaccination coverage in the general population could become better or worse when disclosing both vaccination coverage and disease incidence than when disclosing either piece of information separately.

A significant body of literature in cognitive psychology addresses perception biases of clinical risk (see Refs. [27]–[29] and references therein); however, applications to influenza are limited. Data to characterize the public perception of the benefits of vaccination and its potential trends when epidemiological information is broadcast are currently insufficient. A comprehensive study [25] of seasonal influenza vaccination in the general population was conducted in five European countries over seven influenza seasons, identifying reasons invoked pro and contra vaccination. The chief reasons invoked for getting vaccinated were advice from a family doctor (58.6%) and the perception of influenza as a serious illness (51.9%). The two major reasons for not getting vaccinated were the feeling of not being likely to catch influenza (39.5%) and never having considered the option of being vaccinated (35.8%). More field investigations are needed to explicate these reasons in terms of epidemic variables (e.g., prevalence, coverage, etc.) and build realistic models for the score function 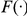. Still, to provide a broad understanding of possible modeling outcomes, here we investigated several examples of how mass media may influence individuals' perceptions of the benefits of vaccination, by reporting infection incidence and vaccination coverage. We note that game theory was particularly suited for our study as it explicitly models risk taking, adaptability and decision making.

In conclusion, the impact of mass-media on social behavior and, implicitly, vaccination coverage is complex. Our game theoretic approach allows for illuminating some of the underlying mechanisms, proposing new perspectives for public health.
